# A Recommendation for the Management of Illness Anxiety Disorder Patients Abusing the Health Care System

**DOI:** 10.1155/2016/6073598

**Published:** 2016-05-25

**Authors:** Mohammad Almalki, Ibrahim Al-Tawayjri, Ahmed Al-Anazi, Sami Mahmoud, Ahmad Al-Mohrej

**Affiliations:** College of Medicine, King Saud Bin Abdulaziz University for Health Sciences, Riyadh, Saudi Arabia

## Abstract

*Introduction*. Illness anxiety disorder (IAD) entails a preoccupation with having a serious, undiagnosed illness in which somatic symptoms are, if present, mild in intensity (American Psychiatric Association, 2013).* Case Report*. This is a case of seventy-three-year-old Saudi man who started visiting the primary health care center around twenty-five years ago. With concerns of having cancer, the patient continuously visited the hospital, costing over $170,000. Throughout this period, the patient has been exposed to extensive unnecessary imaging studies and laboratory tests that have effects on his life in all aspects with such concerns. Five years ago, a family doctor has put an end to that by directing the patient to the right path. The doctor made several actions; most importantly, he directed the patient to a cognitive behavioral therapy which significantly improved a range of hypochondriacal beliefs and attitudes. This patient's case demonstrates the fundamental importance of a proper health system that limits such patients from abusing the health system and depleting the medical resources. Moreover, this case emphasizes the important role of the family physician who can be the first physician to encounter such patients. Thus, proper understanding of the nature of such disorder is a key element for better diagnosis and management.

## 1. Introduction

The American Psychiatric Association in the* Diagnostic and Statistical Manual for Mental Disorders, Fifth Edition (DSM-5)*, categorizes a group of disorders as somatic symptom disorders and other related disorders which were previously known as somatoform disorders in the* Diagnostic and Statistical Manual for Mental Disorders, Fourth Edition, Text Revision (DSM-IV-TR)* [1, 2]. This new category includes a group of disorders which are as follows: somatic symptom disorder, conversion disorder, psychological factors affecting a medical condition, factitious disorder, and other specific and nonspecific somatic symptom disorders [3]. The excessive worries and thoughts of a presumed and nonexisting illness, which was known as hypochondriasis in DSM-4, is now under the umbrella of the somatic symptom disorders [4]. Diagnosis of somatic symptom disorder is met after taking a detailed history with these fears persisting for at least six months despite reassurance after a full medical evaluation [5]. In addition, it has to have at least one somatic symptom that is causing a significant disruption of the patient's life with significant actions and emotions that result in high anxiety level or excessive time consumption [4]. DSM-5 encompasses two types of patients with illness anxiety disorder: care-seeking type and care-avoidant type [1]. Since patients with IAD emanate their distress and anxiety not primarily from the physical complaint itself but rather from his or her anxiety about the meaning, significance, and cause of the complaint, they remain unsatisfied with the reassurance of the physicians [1]. This will cause a huge burden on the resources of the health facility and on its health care providers [1].

## 2. Case Presentation

We present a case of a seventy-three-year-old Saudi man who has started visiting the primary health care center in our institution twenty-five years ago. He has been concerned with having a cancer that would give him only few days to live. At the beginning, the patient was evaluated medically through detailed history and documentation of his symptoms and then a management plan was created accordingly to exclude cancer. Full history, physical examinations, and radiological and pathological investigations were ordered and the results were all negative for cancer. The physician explained the results of the investigations to the patient but he refused them and continued to insist that he had cancer regardless of the results. The patient was then referred to Psychiatry Department to be evaluated but he could not realize that his symptoms might be of a nonorganic cause, either psychological or mental.

The patient continued to visit the general hospital, emergency department, and the primary health care in the institution and was still occupied with the idea of cancer presence. Although the patient was seen by many physicians, the patient was always not satisfied with their conclusions. Eventually a physician reported the case to the department of medical eligibility addressing the issue of continuous primary health care center visits with very variable symptoms, nonconclusive diagnosis, and an unconvinced patient. The department of medical eligibility in the hospital administration took a decision to temporarily limit the patient's file to the psychiatry department to drive the patient to visit the psychiatrist to be evaluated psychologically in order to make his file eligible again. The patient was unhappy at the beginning but he had to visit the psychiatrist. So, an appointment with the psychiatrist was booked and a full psychological and social evaluation was performed by taking a thorough history from the patient. This revealed that the patient fit the criteria of the illness anxiety disorder in which he had a minimum of six months of a persistent belief of having a serious disease which he specifically named. Moreover, this persistent occupation with this belief was disabling and limiting him from having a normal life and thought that his days were counted in this life. Also, a persistent refusal of any medical advice or explanation for his symptoms and fears was neither related to schizophrenia nor related to mood disorders.

A diagnosis of illness anxiety disorder was made despite the patient's strong refusal. The family and social history evaluation also revealed a very low socioeconomic status and similar conditions in the family. Interestingly, two of the patient's daughters had similar reported conditions which could raise the suspicion of the possible genetic predisposition that could be triggered by shared environmental factors between him and his daughters. Following this extensive detailed history and evaluation, the treating psychiatrist contacted the department of medical eligibility to make the patient's file eligible again as promised by them with an unlimited eligibility to all specialties and primary care center. For the past twenty-five years of continuous hospital visiting, he has had almost weekly and even daily visits to the primary care. The patient kept complaining of variable symptoms every week and specifically asking for certain lab tests and radiological studies. Also, he successfully convinced some physicians to order a biopsy for him.

It is reported that physicians in the primary health care center used different techniques of counseling and they applied the biopsychosocial model to overcome the patient's fears but often failed to reach an achievement as the patient continued to complain of different symptoms and became a burden on the physician's clinic time and the hospital resources. It is also reported that the patient was extremely difficult to handle and convince. In the absence of institution guidelines to deal with such cases, all physicians tended to yield to the patient's persistent complaints and accepted his demands and fulfilled his desires by requesting whatever investigations the patient asked for. Over the years, the patient's investigations have cost approximately $178200 due to the system negligence to such cases. The patient's most requested investigations were mainly invasive radiological studies specifically abdomen and pelvis CT (18 times) and wide range of other invasive and noninvasive studies like chest CT (11 times), brain CT (7 times), chest MRI (4 times), lumbar spine MRI (6 times), and other studies. It is important here to put emphasis on the potential harms of radiation from radiological studies that are frequently requested by the patient in the absence of clear guidelines to be followed by practitioners for that group of patients. In addition, the patient asked for a variety of lab tests including renal profile, parathyroid hormone, complete blood count, estimated glomerular filtration rate, coronary risk profile, 25-hydroxy vitamin D, thyroid stimulating hormone, prostate specific antigen, free T4 level, and other very wide range of lab tests. Moreover, the patient underwent several biopsies like renal biopsy, prostate biopsy, gastric biopsy, and other different types of biopsies.

The patient was abusing the system in which resources and time were wasted. The patient kept overstepping each barrier by the hospital's staff to minimize his burden on the hospital by manipulating staff and deceiving and misleading them by giving incorrect information. Also, he was targeting junior practitioners and disturbing them and even threatened to sue the administration if restricted from health care. Five years ago, an experienced board-certified family physician was shocked when he looked at the patient's file and decided to put an effort to solve this problem by taking some actions. First action considered, after getting the approval from the family medicine department, was to limit the patient's primary care visits to only one clinic and to refer him to the supervising physician himself. Then, building strong and effective patient rapport based on trust and honesty was initiated. After negotiations, the patient agreed to enroll in cognitive behavioral therapy (CBT) for six sessions ninety minutes each. The patient was advised to have a scheduled sleeping time, ensure healthy eating habits with regular physical exercise, be involved in social activities, stay away from stressors like searching web for symptoms, and avoid TV health shows and health magazines during active cycles of disorder. After that, there was an agreement with the patient not to disturb the clinic and he would get to see the physician regularly for checkups every three months.

After five years of implementing the new rules, the patient is still visiting the clinic for regular follow-up and sometimes he breaks the rules and comes regularly to the clinic especially in active cycles of the disorder. On following the patient's condition in the last five years, it is noticed that there is a dramatic decrease in the total financial cost due to the relative decreasing number of visits, less time spent on arguing with the patient, and the filtration of his requests. Moreover, there is a remarkable improvement in the patient's condition because of CBT and relative compliance to health advice. We think that this rare case of extremely persisting illness anxiety is worth reporting because the patient managed somehow to escape attention and made a huge financial burden on hospital resources in the absence of clear guidelines in such conditions. Also, this case provides useful insight for future guidelines development.

## 3. Discussion

Because they consider themselves medically ill, individuals with IAD are usually encountered in medical rather than in mental health setting. Moreover, they show elevated rates of medical utilization by consulting multiple physicians for the same complaint and obtaining repeatedly negative diagnostic test results [1]. They do that by manipulating the physicians to order them diagnostic tests and imaging studies, as for this patient who has cost the hospital more than $170,000 through ordering more than thirty CTs and ten MRIs in addition to lab workups over the course of twenty-five years. As a result, patients with IAD will have a burden on the institution's budget as well as their increased risk of adverse health events [6].

Physicians in the medical setting mostly fail to early recognize and deal with such situations leading to excessive use of unnecessary diagnostic tests and imaging studies [1]. It was estimated that 10% to 20% of the US medical budget is spent on patients who somatize or have hypochondriacal concerns [7]. Because those patients do not respond to appropriate medical reassurance or negative diagnostic tests, the physician's attempts at reassurance and symptom palliation generally do not alleviate [1]. As a result, this can lead to physician shopping, bouncing from one clinic to another seeking for reassurance that cannot be delivered through the conventional ways [1]. Therefore, they will deplete medical institution's resources and physician's time and efforts [6]. Such behavior will leave most physicians frustrated and puzzled on how to deal with such patients. Therefore, one of the most important points in the management of illness anxiety disorder is to avoid this escalation of mutual mistrust by establishing a good therapeutic relationship with the patient, regardless of the specific treatment modality that will be used [8].

Another important aspect of this problem is the potential harms that can happen to the patients by exposing themselves to unnecessary, extensive, and invasive imaging studies which increase their risk of adverse health events [9]. As for this patient, he was exposed to more than twenty-five CT scans, collectively reaching 268 mSv, increasing his excess cancer relative risk up to two hundred fifty more than general population [9]. In addition, illness concerns assume a prominent place in the individual's life, affecting daily activities, social life, and may eventually result in invalidism. Illness becomes a central feature of the individual's identity, a frequent topic of social discourse, and a characteristic response to stressful life events [1].

After over twenty-five years of seeking medical attention, a family doctor has put an end to this by directing the patient to the right path. The doctor took several actions like directing the patient to a cognitive behavioral therapy for six sessions ninety minutes each. The six sessions directed towards the cognitive and perceptual mechanisms thought to underlie the disorder appear to significantly improve a range of hypochondriacal symptoms, beliefs, and attitudes [10]. Furthermore, it is important to keep in mind that IAD is a chronic disease and that CBT sessions should be followed by follow-up sessions which are considered as booster sessions [10].

In general, this case report shows the full course of an IAD patient. It shows how the symptoms started and the huge burden of it on the patient himself, his family, medical practitioners, and the hospital. Moreover, it shows the course of the treatment and how it has helped the patient to improve significantly, although there are still active cycles of the disorder, which can be reduced by applying further booster CBT sessions. Finally, this case report fails to specifically mention the frequency of the active cycles and the severity of them.
